# Increase in methicillin-susceptible *Staphylococcus aureus* bloodstream infections in Switzerland: a nationwide surveillance study (2008–2021)

**DOI:** 10.1007/s15010-023-01980-6

**Published:** 2023-02-02

**Authors:** Luzia Renggli, Michael Gasser, Niccolò Buetti, Andreas Kronenberg, the Swiss Centre for Antibiotic Resistance

**Affiliations:** 1 Swiss Centre for Antibiotic Resistance (ANRESIS), Institute for Infectious Diseases, University of Bern, Bern, Switzerland; 2 Graduate School for Health Sciences (GHS), University of Bern, Bern, Switzerland; 3 Infection Control Programme and WHO Collaborating Centre on Infection Prevention and Control and Antimicrobial Resistance, Geneva University Hospitals, Geneva, Switzerland; 4 UMR 1137, IAME, INSERM, Université de Paris, 75018 Paris, France

**Keywords:** Methicillin-susceptible *Staphylococcus aureus*, Methicillin-resistant *Staphylococcus aureus* bloodstream infections, Bacteraemia, Switzerland

## Abstract

**Purpose:**

An increasing burden of *Staphylococcus aureus* bloodstream infections (BSI), despite a decrease in the percentage of methicillin-resistant *S. aureus* (MRSA), was described recently in other European countries. The main aim of this study was to analyse recent temporal trends of *S. aureus*, methicillin-susceptible *S. aureus* (MSSA) and MRSA BSI for Switzerland as well as the different linguistic regions within Switzerland. An additional aim was to estimate potential differences among patient-based and epidemiological risk factors.

**Methods:**

A retrospective observational study was conducted in Switzerland over a period of 14 years (2008–2021)*.* Trends in *S. aureus*, MSSA and MRSA BSI were analysed by applying linear regression models.

**Results:**

*Staphylococcus aureus* BSI increased by + 30% from 19.7 to 25.6 cases per 100,000 inhabitants between 2008 and 2021 (*P* < 0.01) in Switzerland. Thereof, MSSA increased by + 37% from 17.8 to 24.4 cases per 100,000 inhabitants (*P* < 0.01). MRSA decreased from 1.9 to 1.2 cases per 100,000 inhabitants (*P* < 0.01), which was driven by decreasing incidence in the French-speaking region. MSSA BSI increased significantly (*P* < 0.01) in both linguistic regions. A further stratification revealed that incidence increased the most in male patients of the age group ≥ 80 years of the German-speaking region.

**Conclusion:**

The increasing health burden of MSSA BSI in Switzerland indicates that not only proportions of resistant microorganisms but also total BSI incidences should be monitored. In addition, data stratification revealed that the increase was mainly driven by an increasing incidence in elderly males of the German-speaking region.

**Supplementary Information:**

The online version contains supplementary material available at 10.1007/s15010-023-01980-6.

## Introduction

Bloodstream infections (BSI) are associated with increased mortality, with *Staphylococcus aureus* being one of the most common pathogens [1–4]. An increasing burden of absolute numbers of *S. aureus* BSI despite a decrease in the percentage of methicillin-resistant *S. aureus* (MRSA) was recently described for the European Union (EU) and European Economic Area (EEA) between 2005 and 2018 [5]. Switzerland is not an EU or EEA country and thus was not part of this project. In two earlier epidemiologic analyses of *S. aureus* BSI in Switzerland (2008–2014), a stable incidence was reported [6], with a decreasing percentage of MRSA (2004–2014)[7]. For the incidence of invasive MRSA infections, decreasing trends were described more recently for Switzerland overall, with different trends between the Swiss linguistic regions [8]. In addition to location in the French and Italian linguistic regions of Switzerland, Olearo et al*.* reported inpatient status and elderly age as risk factors for MRSA (compared with MSSA) [7]. Further studies identified older age and male sex as risk factors for *S. aureus* BSI [4, 9]. To target possible future prevention strategies, a timely analysis of the epidemiological situation in Switzerland is needed.

The main aim of this study was to analyse recent temporal trends of *S. aureus*, MSSA and MRSA BSI for Switzerland as well as the different linguistic regions within Switzerland. An additional aim was to estimate potential differences among patient-based and epidemiological risk factors for MRSA or MSSA [4, 7, 9].

## Methods

### Design and study population

A retrospective observational study was conducted in Switzerland over a period of 14 years (2008–2021). *S. aureus* BSI from 70 acute care hospitals that reported data in 2008 and 2021 and for more than half of the years within this period to the Swiss Centre for Antibiotic Resistance (ANRESIS) database were included (see map Supplementary Fig. 1).

**Fig. 1:**
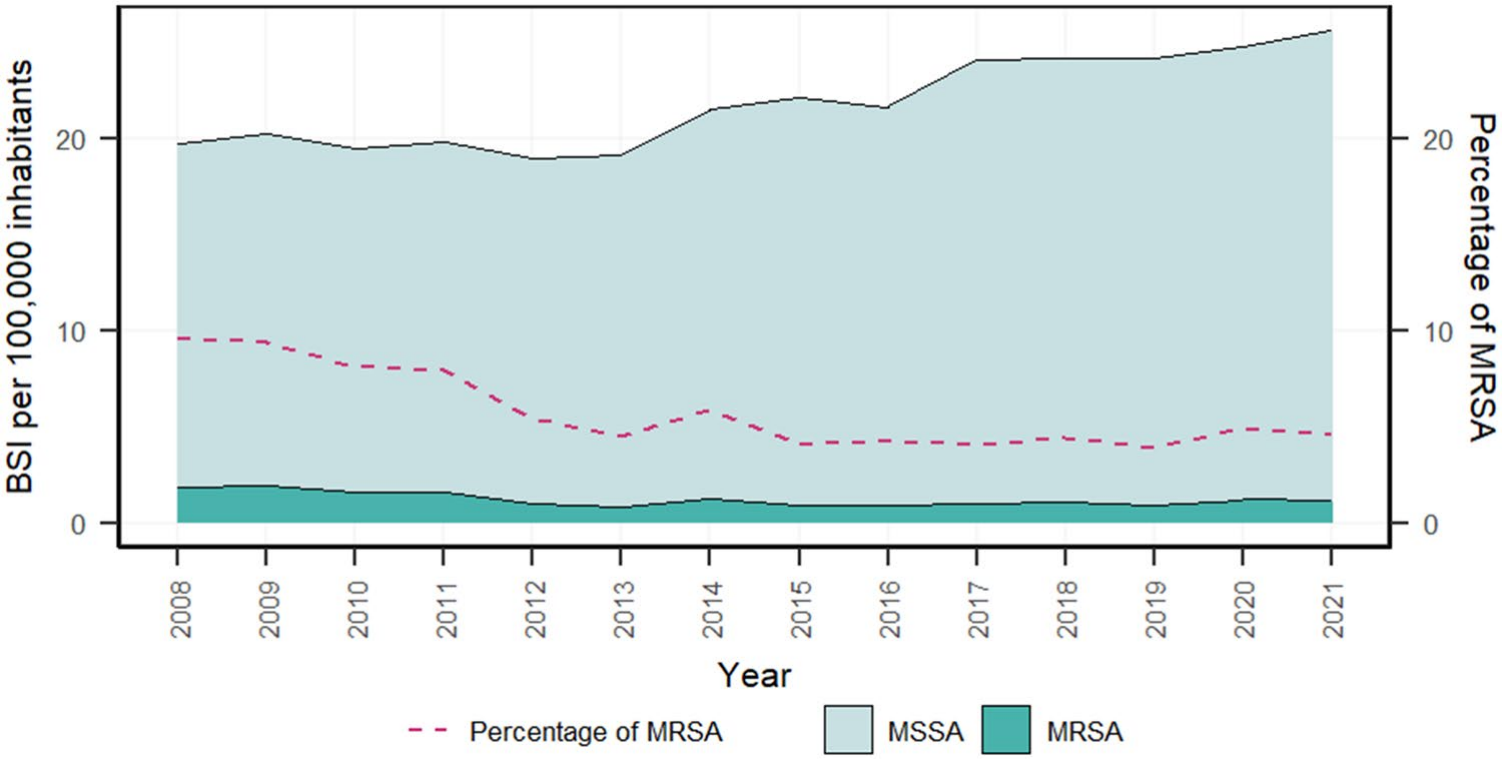
Incidence of MSSA and MRSA bloodstream infections (BSI) and percentage of MRSA among *S. aureus* BSI, Switzerland (2008–2021). BSI, bloodstream infections; MSSA, methicillin-susceptible *Staphylococcus aureus*; MRSA, methicillin-resistant *Staphylococcus aureus*

### Data collection and processing

Data on *S. aureus* BSI were obtained from the ANRESIS database [10]. The participating laboratories are accredited by national authorities. During the study period, guidelines for antibiotic susceptibility testing changed from CLSI to EUCAST guidelines; however, breakpoints for methicillin did not change. MRSA BSI was defined as a blood culture containing *S. aureus* resistant to at least one of the following antibiotics: methicillin, oxacillin, flucloxacillin, or cefoxitin. The first blood culture with resistance testing for at least one of these antibiotics per patient per year was considered.

The number of cases per 100,000 inhabitants was extrapolated by the yearly bed-days covered by ANRESIS (coverage in Switzerland overall in 2014, 53%; French-speaking region, 75%; and German-speaking region, 51%).

In the main analysis, data were stratified by linguistic region (German-speaking *versus* French-speaking region), patient age group (< 2 years, 2–24 years, 25–49 years, 50–64 years, 65–79 years and ≥ 80 years), sex (male or female) or hospital unit (ICU *versus* outpatient department *versus* other departments). The number of inhabitants was used as the denominator except for the analysis of the hospital unit, as inhabitants cannot be allocated to a hospital unit. For Switzerland overall, the incidence in 2008 and 2021 was additionally calculated using bed-days.

An explanatory analysis was performed differing between community- and hospital-onset BSI. Samples taken within the first 48 h after admission were considered community-onset BSI. As this information was not available for 38% of the samples, they could not be included. Therefore, the absolute number of BSI was analysed.

Two further explanatory analyses were performed with datasets for methicillin-susceptible *S. aureus* that used non-blood specimens. Samples from bone or prosthetic joints containing *S. aureus* were used as surrogate marker for bone and joint infections. Moreover, samples from wound samples and biopsies were used as surrogate markers for skin and soft tissue infections.

### Statistical analysis

The statistical plan comprised three steps. First, temporal trends in *S. aureus*, MSSA and MRSA BSI per 100,000 inhabitants for Switzerland overall and for each linguistic region were analysed by applying a linear regression model for Switzerland overall and for each linguistic region separately. Second, a logistic regression model was used to analyse the proportions of MRSA among *S. aureus* BSI over time. Third, to analyse trends in MSSA bone and joint infections as well as MSSA skin and soft tissue infections, a linear regression model was applied for each linguistic region. The results were considered significant when the model fit an R-square above 0.4 and the p value of the explanatory variable was below 0.05. All analyses were performed using R software (version 4.1.2., R Core Team, Vienna, Austria).

## Results

During the study period, 17,012 blood cultures containing *S. aureus* were reported to ANRESIS (Supplementary Table 1). *S. aureus* increased by + 30% from 19.7 to 25.6 cases per 100,000 inhabitants between 2008 and 2021 in Switzerland (Table 1). Thereof, MSSA BSI increased by + 37% from 17.8 to 24.4 cases per 100,000 inhabitants (*P* < 0.01), while MRSA BSI decreased from 1.9 to 1.2 cases (*P* < 0.01). This resulted in a significant (*P* < 0.01) decrease in the proportion of MRSA on *S. aureus* BSI over time (Fig. 1).

**Table 1:** Temporal course of *Staphylococcus aureus* bloodstream infections (BSI): total, MSSA, MRSA and the percentage of MRSA among *S. aureus* BSI, Switzerland (2008–2021)

*S. aureus* BSI	BSI per 100,000 inhabitants				BSI per 1,000 bed-days		
	2008	2021	Change (%)	Trends	2008	2021	Change (%)
Total	19.7	25.6	30	↑ (*P* < 0.01)	0.277	0.412	49
MSSA	17.8	24.4	37	↑ (*P* < 0.01)	0.251	0.393	57
MRSA	1.9	1.2	− 37	↓ (*P* < 0.01)	0.026	0.019	− 28
% MRSA	9.5%	4.6%	− 52	↓ (*P* < 0.01)	9.5%	4.6%	− 52

*ns* not significant, *BSI* bloodstream infections, *MSSA* methicillin-susceptible *Staphylococcus aureus*, *MRSA* methicillin-resistant *Staphylococcus aureus*

**Fig. 2:**
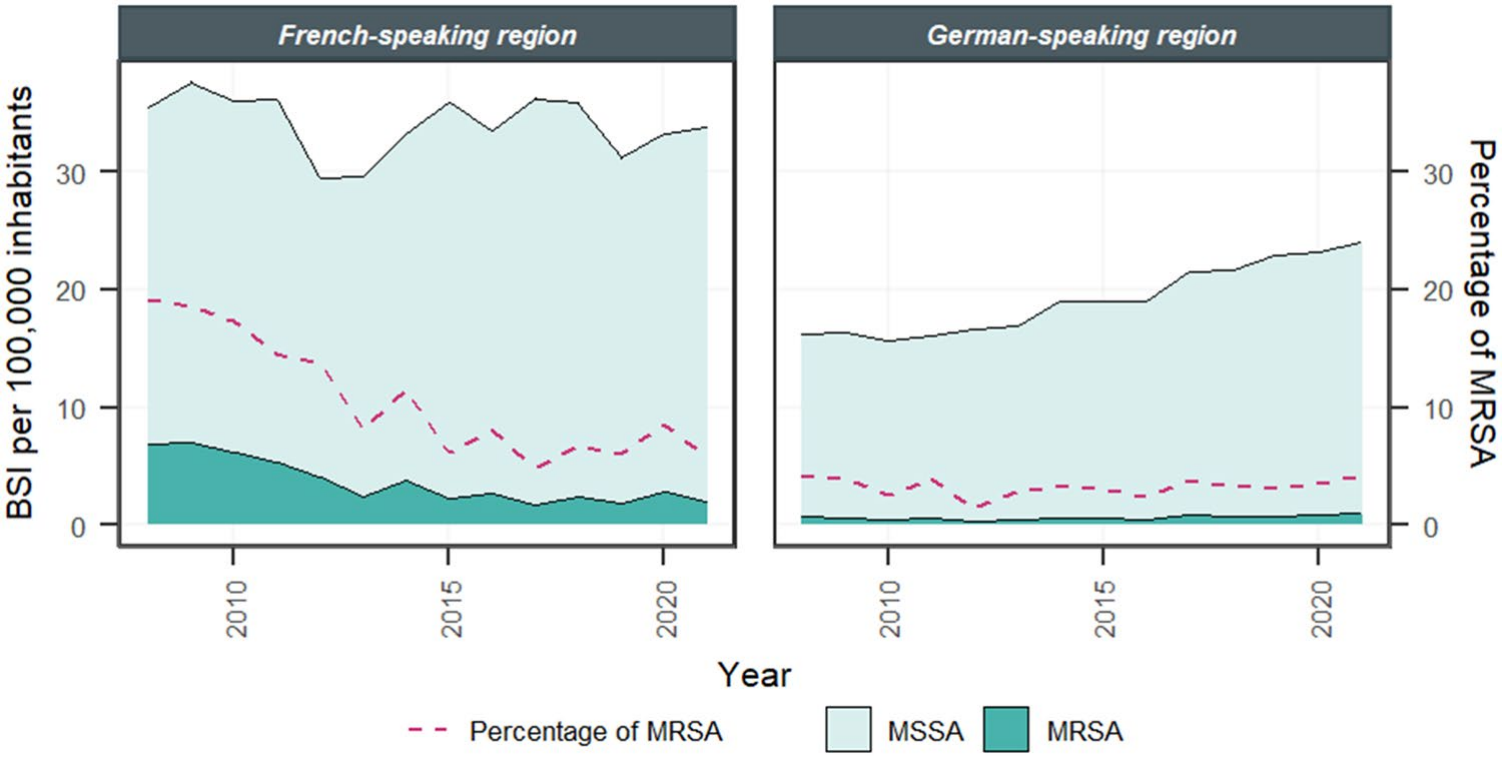
Incidence of methicillin-susceptible (MSSA) and methicillin-resistant *Staphylococcus aureus* (MRSA) bloodstream infections (BSI) and percentage of MRSA among *S. aureus* BSI in the different linguistic regions of Switzerland (2008–2021). BSI, bloodstream infections; MSSA, methicillin-susceptible *Staphylococcus aureus*; MRSA, methicillin-resistant *Staphylococcus aureus*

Temporal trends in MRSA BSI differed between the linguistic regions within Switzerland: the incidence of MRSA BSI decreased significantly in the French-speaking region (*P* < 0.01), whereas a slight increase (*P* < 0.01) was observed in the German-speaking region, although it remained at a low level (Fig. 2, Supplementary Table 2).

The number of MSSA BSI increased significantly (*P* < 0.01) in both linguistic regions, but the increase was more pronounced in the German-speaking region (+ 58%) than in the French-speaking region (+ 13%). A significant increase was observed for MSSA bone and joint infections in the German-speaking region (*P* < 0.01), while the increase was not significant in the French-speaking region (Supplementary Table 6, Supplementary Fig. 6). Inpatient skin and soft tissue infections did not change significantly in the German-speaking region and decreased (P < 0.01) in the French-speaking region (Supplementary Fig. 7).

**Fig. 3:**
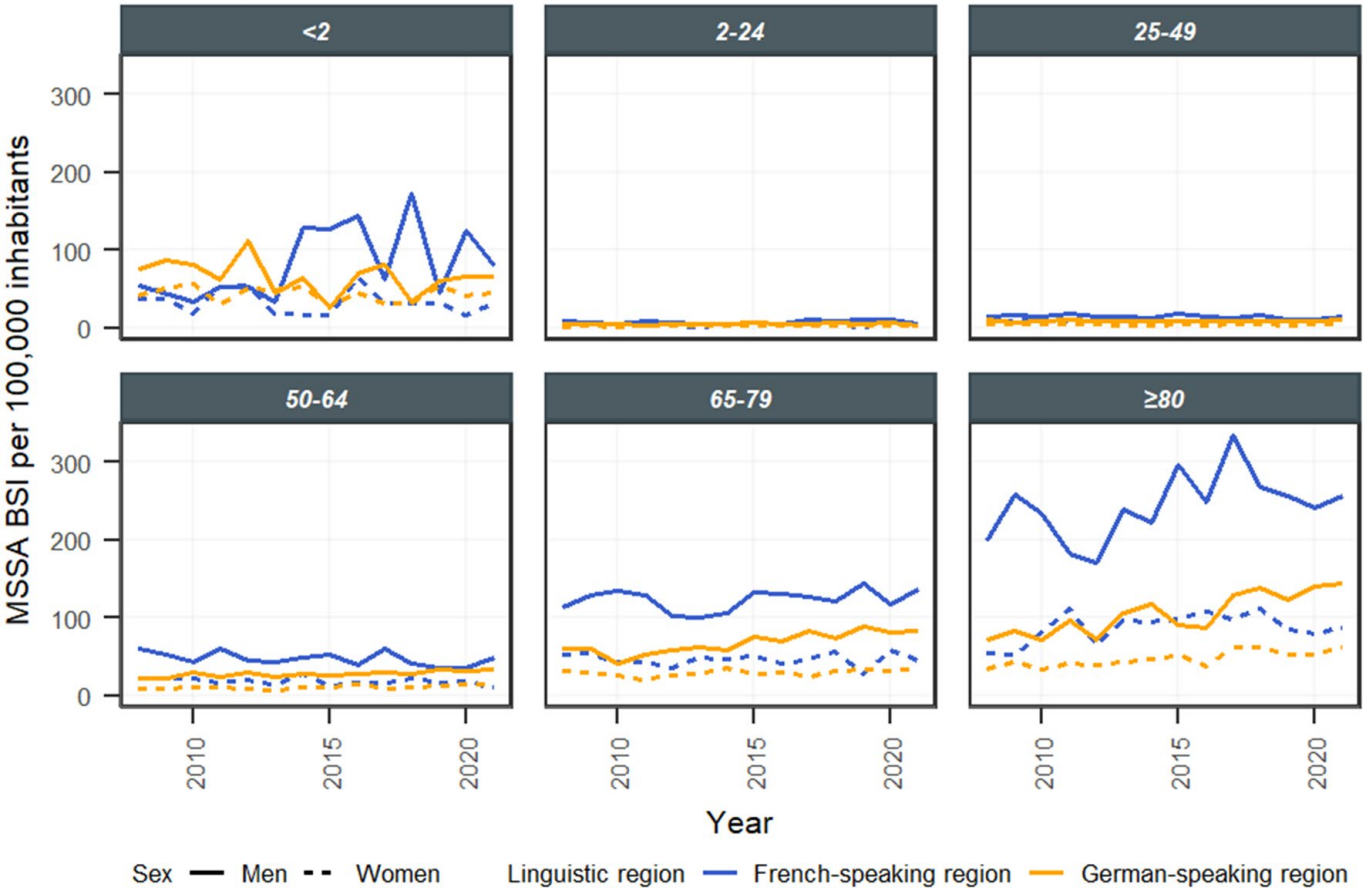
Temporal course of bloodstream infections (BSI) caused by methicillin-susceptible *Staphylococcus aureus* (MSSA) in the different linguistic regions stratified by age group and sex, Switzerland (2008–2021)

Further stratifications by age and sex revealed that the increase in incidence was mainly driven by patients of the German-speaking region in the age categories of 50–64 years, 65–79 years and ≥ 80 years (Fig. 3, Supplementary Table 3). The proportional increase was highest in the age category of ≥ 80 years, followed by 50–65 years and 65–79 years, with a stronger increase in males than in females.

MSSA BSI increased in all hospital types (university *versus* non-university ≥ 200 beds *versus* non-university < 200 beds) in both linguistic regions. The variability in the proportional increase was high, especially among non-university hospitals with less than 200 beds (Supplementary Fig. 5).

The increase in absolute MSSA BSI numbers was higher in the outpatient department than in the ICU and the other non-ICU departments (Supplementary Fig. 4). The sub-analysis with the restricted dataset of samples labelled with the hospitalisation date showed a higher increase in community-onset MSSA BSI than in hospital-onset MSSA BSI (Supplementary Table 5, Supplementary Fig. 8).

## Discussion

The health burden of *S. aureus* BSI increased in Switzerland between 2008 and 2021: + 30% from 19.7 to 25.6 cases per 100,000 inhabitants caused by an increasing incidence of MSSA BSI. Conversely, the incidence of MRSA BSI was slightly decreasing, which resulted in a decreased percentage of MRSA. These trends mirror the situation in most EU or EEA countries [5]. The incidence of *S. aureus* BSI in Switzerland was lower than that in northern Europe, although higher than that in Spain (Finland, 2015: 35.2 cases/100,000 inhabitants [11]; Denmark, mean for 2008–2015: 24.9 cases/100,000 inhabitants [12]; Spain, mean for 2013–2017: 9.3 cases/100,000 inhabitants [12]).

Surprisingly, the increasing trend in MSSA BSI was more pronounced in the German-speaking region than in the French-speaking region. The same patterns were observed for bone and joint infections. During the same time period, the number of surgical procedures for implementing or changing pacemakers increased in the German-speaking region while remaining constant in the French-speaking region [13] and may thus partly explain these regional differences, as devices are a common source of *S. aureus* BSI, and previous hospitalisation is a known risk factor [14]. In future studies, the relationship between device placements and *S. aureus* BSI over time considering possible changes in perioperative procedures must be analysed. Another possible reason for these regional discrepancies of MSSA may be that prevention measures to control MRSA have been implemented more in the French-speaking region [15, 16] since MRSA incidence was more than six-fold higher in the French-speaking compared to the German-speaking region to begin of the study period (discussed further below). This may have impacted the spread of MSSA. Superficial injuries are another important source of BSI [17], but MSSA skin and soft tissue infections did not increase during the study period. However, the incidence of MSSA skin and soft tissue infections mirrored the differences between the linguistic regions, with no significant trend in the German-speaking region and a significant decrease in the French-speaking region.

Finer stratifications revealed that increases in MSSA BSI as well as MSSA bone and joint infections were mainly driven by elderly males of the German-speaking region. This is in line with observations in most EU or EEA countries where the increase in *S. aureus* BSI was also more pronounced in males and in elderly individuals [5, 18]. Among others, age and male sex are well-described risk factors for *S. aureus* BSI [4, 9]. Although the elderly population was growing during the study period, this risk factor by itself may not explain the observed increase in incidence, as age-stratified populations were considered denominators. Other known risk factors for *S. aureus* BSI, such as diabetes mellitus and immunosuppression, probably increased slightly during the study period and could at least in part explain the overall increase [4, 19]. However, we were not able to quantify these factors, and we do not expect relevant differences between the French- and German-speaking regions. Further hypothetical explanations, such as the association between changes in smoking habits and *S. aureus* colonisation, are controversial [9].

The increase in MSSA BSI was higher in the outpatient department than in the ICU department as well as in community-onset MSSA BSI than in the hospital-onset MSSA BSI. The finding that an increase in MSSA BSI was likely to be driven by community-onset BSI was not in contrast to the previous hypothesis of increasing device placements as a source of *S. aureus* BSI, because community-onset was defined as a microbiology sample taken within 48 h after admission. As early prosthetic joint infection is defined as occurring within 3 months after surgery and the onset of *S. aureus* infection after hip or knee arthroplasty was described to have a median of 28 days (interquartile range 18–63 days) [20], these infections are likely to be defined as community-onset. Recent studies have reported an increasing incidence and virulence of the *S. aureus* CC398 lineage, a strain that originates from livestock, in the community setting [21]. Further studies need to investigate whether a more virulent and transmissible strain is the reason behind the increasing MSSA BSI incidence in Switzerland.

The decrease in MRSA BSI on a national level was caused solely by decreasing incidence in the French-speaking region, as the incidence even increased slightly in the German-speaking region. However, in 2008, the incidence of MRSA BSI was higher in the French-speaking region and converged during the study period to the levels of the German-speaking region, which may be explained by health care measures and an evolution of MRSA towards clones with reduced fitness [22–25]. These regionally differing trends for MRSA BSI were consistent with previous results [7, 8].

This study has some limitations, mainly due to its observational nature. First, we performed an epidemiological study using data from a microbiological surveillance system: several clinical individual patient data (e.g., baseline comorbidities, reasons for admission, and source of BSI) were not available. Although hospital coverage (53%) was achieved at the national level, no hospital from the Italian-speaking region was included due to inconsistent reporting. However, all Swiss university hospitals were included in this analysis. In addition, the hospitalisation date was available for only 62% of the samples, thus reducing the validity of this explanatory analysis. As the geographical distribution of samples with the hospitalisation data available was uneven and whether the labelling was performed thoroughly was unknown, the results of this analysis need to be interpreted with caution. However, the results seem plausible, as trends in analyses comparing outpatient, ICU and other departments could be confirmed.

The main strength of our study is the extensive data collection covering 14 years and all university hospitals in Switzerland. In addition, the analysis of Swiss data allowed stratification into different linguistic and sociocultural regions due to the country's heterogeneity.

In conclusion, the increasing health burden of MSSA bloodstream infections (BSI) in Switzerland indicates that surveillance should consider not only proportions of resistant microorganisms but also, more importantly, incidence rates of BSI. In addition, a stratification by linguistic region, sex and age group revealed that the increase in MSSA BSI was heterogeneous and mainly driven by an increasing incidence in elderly males of the German-speaking region. To formulate appropriate regionally targeted measures for this population, further investigations examining the underlying reasons for the growing incidence are needed.

## Supplementary Information

Below is the link to the electronic supplementary material.

Supplementary file1 (DOCX 1003 kb)

## Data Availability

All data can be made available upon request to the corresponding author.
