# Supplementary material for: Increase in methicillin-susceptible Staphylococcus aureus bloodstream infections in Switzerland: a nationwide surveillance study (2008–2021)
Source: Infection. 2023 Feb 2;51(4):1025–31. doi: 10.1007/s15010-023-01980-6 (PMC10352440; doi:10.1007/s15010-023-01980-6)
Supplement: Supplementary file 1 — Supplementary file1 (DOCX 1003 kb) [file 15010_2023_1980_MOESM1_ESM.docx]

## Increase in methicillin-susceptible *Staphylococcus aureus* bloodstream infections in Switzerland: a nationwide surveillance study (2008-2021)

Renggli L^1,2^, Gasser M^1^, Buetti N ^3,4^, Kronenberg A^1^ and the Swiss Centre for Antibiotic Resistance

1. Swiss Centre for Antibiotic Resistance (ANRESIS), Institute for Infectious Diseases, University of Bern, Bern, Switzerland

2. Graduate School for Health Sciences (GHS), University of Bern, Bern, Switzerland

3. Infection Control Programme and WHO Collaborating Centre on Infection Prevention and Control and Antimicrobial Resistance, Geneva University Hospitals, Geneva, Switzerland

4. UMR 1137, IAME, INSERM, Université de Paris, 75018, Paris, France

Corresponding author: Luzia Renggli, [luzia.renggli@unibe.ch](mailto:luzia.renggli@unibe.ch)

Journal: Infection

# Supplementary Material


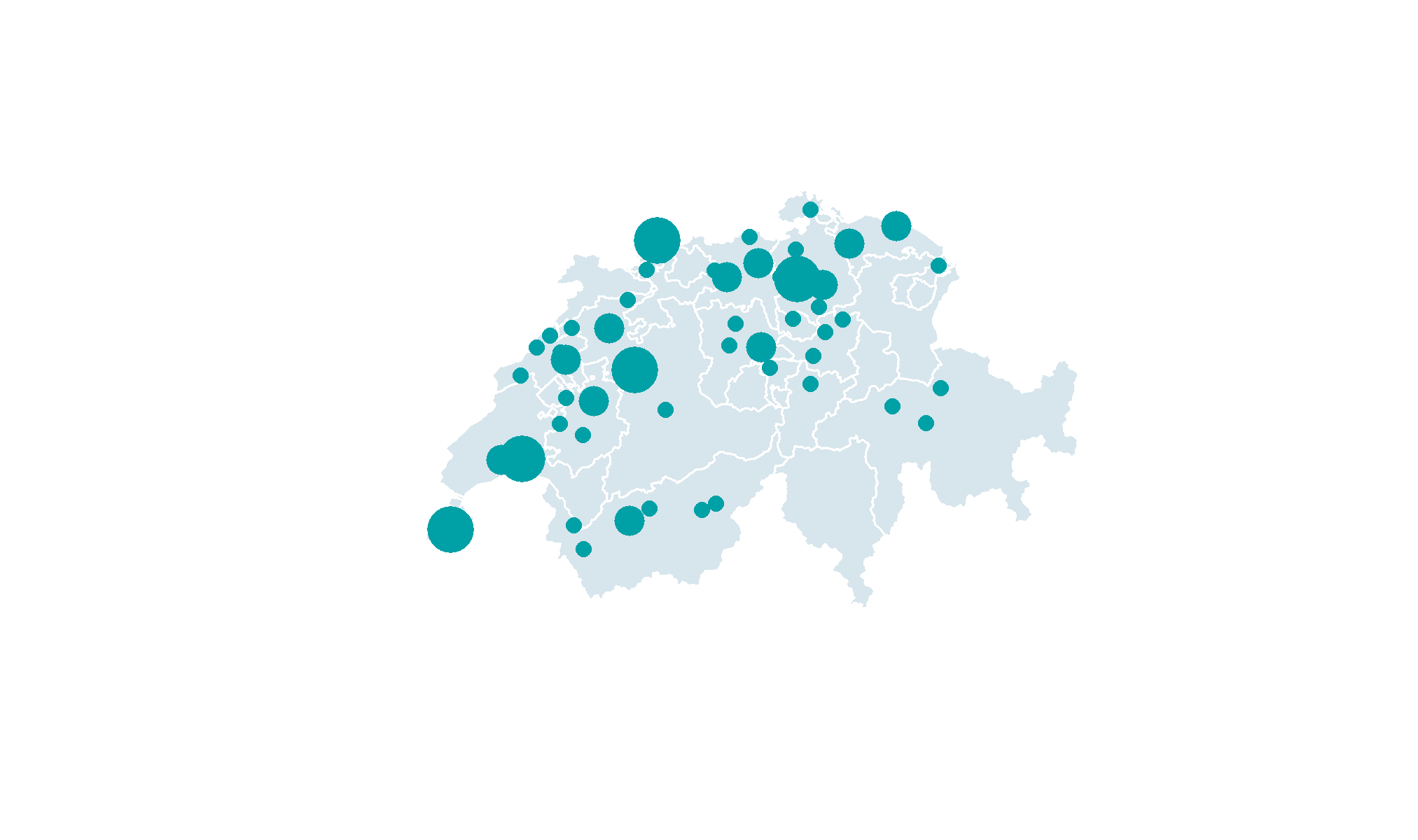


Supplementary Figure 1: Swiss map with the included hospitals. The hospital size is visualised by the size of the points (university *versus* non-university ≥ 200 beds *versus* non-university < 200 beds).

Supplementary Table 1: Sample characteristics of all positive blood cultures included in the study

|  |  | **Proportion during total study period** |
| --- | --- | --- |
|  |  | **(N isolates=17,012)** |
| MRSA |  | 5.6% |
| French-speaking region | | 32.3% |
| Hospital type: | university | 43.8% |
|  | non-university ≥200 beds | 30.1% |
|  | non-university <200 beds | 26.1% |
| Department: | ICU | 8.9% |
|  | outpatient | 35.8% |
|  | other departments | 55.3% |
| Men |  | 66.5% |
| Age | <2 | 2.6% |
|  | 2-24 | 5.4% |
|  | 25-49 | 13.3% |
|  | 50-64 | 22.0% |
|  | 65-79 | 34.2% |
|  | ≥80 | 22.4% |

N, number; MRSA, methicillin-resistant *S. aureus*; ICU, intensive care unit

Supplementary Table 2: Temporal course of *S. aureus*, MSSA and MRSA BSI stratified by linguistic region in percentage and cases per 100,000 inhabitants, Switzerland, 2008-2021

| ***S. aureus* BSI** | **French-speaking region** | | | | **German-speaking region** | | | | | |
| --- | --- | --- | --- | --- | --- | --- | --- | --- | --- | --- |
|  | **2008** | **2021** | **Change** | **Trends** | **2008** | **2021** | **Change** | | | **Trend** |
| Total | 24.22 | 23.44 | -3% | ns | 18.06 | 26.76 | | +48% | ↑ (P<0.01) | |
| MSSA | 19.56 | 22.09 | +13% | ↑ (P<0.01) | 17.33 | 25.64 | | +48% | ↑ (P<0.01) | |
| MRSA | 4.66 | 1.35 | -71% | ↓ (P<0.01) | 0.72 | 1.11 | | +54% | ↑ (P<0.01) | |
| % MRSA | 19.24% | 5.77% | -70% | ↓ (P<0.01) | 4.01% | 4.16% | | +4% | ns | |

ns, not significant; BSI, bloodstream infections; MSSA, methicillin-susceptible *S. aureus*; MRSA, methicillin-resistant *S. aureus*

Supplementary Table 3: Temporal change in bloodstream infections caused by methicillin-susceptible *Staphylococcus aureus* stratified by linguistic region, sex and age category in percentage and cases per 100,000 inhabitants (2008-2021)

| **Sex** | **Age** | **French-speaking region** | | | **German-speaking region** | | |
| --- | --- | --- | --- | --- | --- | --- | --- |
|  | **category** | **2008** | **2021** | **Change** | **2008** | **2021** | **Change** |
| Men | <2 | 36.52 | 55.12 | +51% | 84.94 | 73 | -14% |
|  | 2-24 | 5.78 | 3.5 | -39% | 6.1 | 4.41 | -28% |
|  | 25-49 | 10.76 | 9.65 | -10% | 11.37 | 11.73 | +3% |
|  | 50-64 | 41.25 | 34.25 | -17% | 24.63 | 37.15 | +51% |
|  | 65-79 | 77.03 | 98.08 | +27% | 66.76 | 90.67 | +36% |
|  | ≥80 | 133.15 | 174.79 | +31% | 79.99 | 160 | +100% |
| Women | <2 | 25.63 | 23.21 | -9% | 47.69 | 51.13 | +7% |
|  | 2-24 | 4.02 | 3.22 | -20% | 2.3 | 4.25 | +85% |
|  | 25-49 | 4.94 | 6.43 | +30% | 6.38 | 5.39 | -16% |
|  | 50-64 | 15.21 | 8.21 | -46% | 10.33 | 15.73 | +52% |
|  | 65-79 | 35.27 | 31.94 | -9% | 34.77 | 40.5 | +16% |
|  | ≥80 | 35.98 | 58.06 | +61% | 39.02 | 69.42 | +78% |

Supplementary Table 4: Temporal changes in MSSA bone and joint infections and MSSA skin and soft tissue infections stratified by percentage and cases per 100,000 inhabitants (2008, 2021)

| **MSSA** | **French-speaking part** | | | | **German-speaking part** | | | |
| --- | --- | --- | --- | --- | --- | --- | --- | --- |
|  | **2008** | **2021** | **Change** | **Trend** | **2008** | **2021** | **Change** | **Trend** |
| Bone and joint | 1.51 | 1.97 | +31% | ns | 1.78 | 3.37 | +88% | ↑ (P < 0.01) |
| Skin and soft tissue | 114.61 | 87.34 | -24% | ↓ (P<0.01) | 98.31 | 76.00 | -22% | ns |

MSSA, methicillin-susceptible *S. aureus*; ns, not significant

Supplementary Table 5: Temporal changes in *S. aureus*, MSSA and MRSA BSI stratified by community-onset *versus* hospital-onset in percentage and absolute number of cases (2008, 2021)

| ***S.aureus* BSI** | **Community-onset** | | | **Hospital-onset** | | |
| --- | --- | --- | --- | --- | --- | --- |
|  | **2008** | **2021** | **Change** | **2008** | **2021** | **Change** |
| Total | 447 | 660 | +48% | 283 | 319 | +13% |
| MSSA | 421 | 637 | +51% | 22 | 305 | +37% |
| MRSA | 26 | 23 | -11% | 61 | 14 | -77% |

BSI, bloodstream infection; MSSA, methicillin-susceptible *Staphylococcus aureus*; MRSA, methicillin-resistant *Staphylococcus aureus*


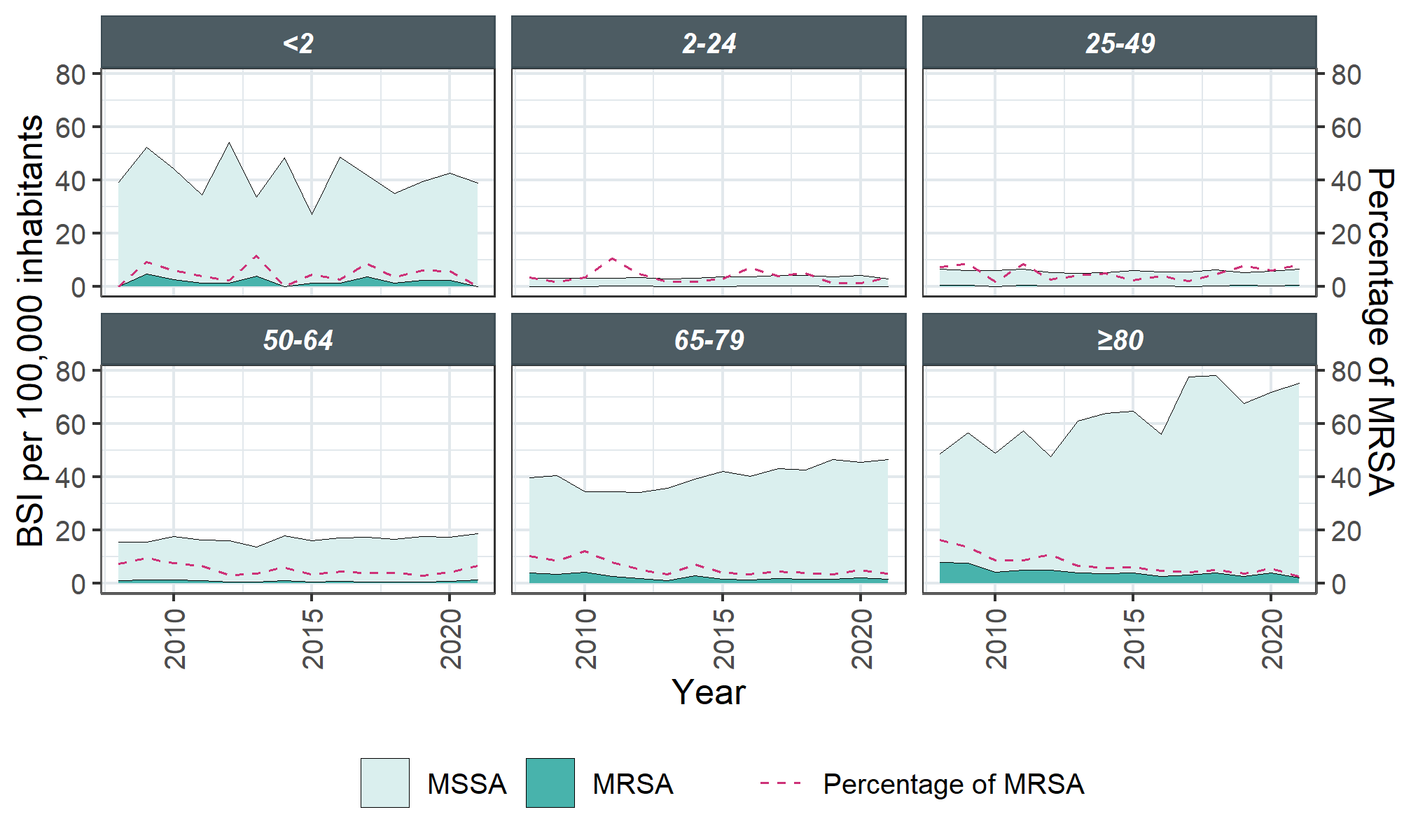


Supplementary Figure 2: Incidence in MSSA and MRSA bloodstream infections (BSI) and percentage of MRSA among *S. aureus* BSI stratified by age category (2008-2021)


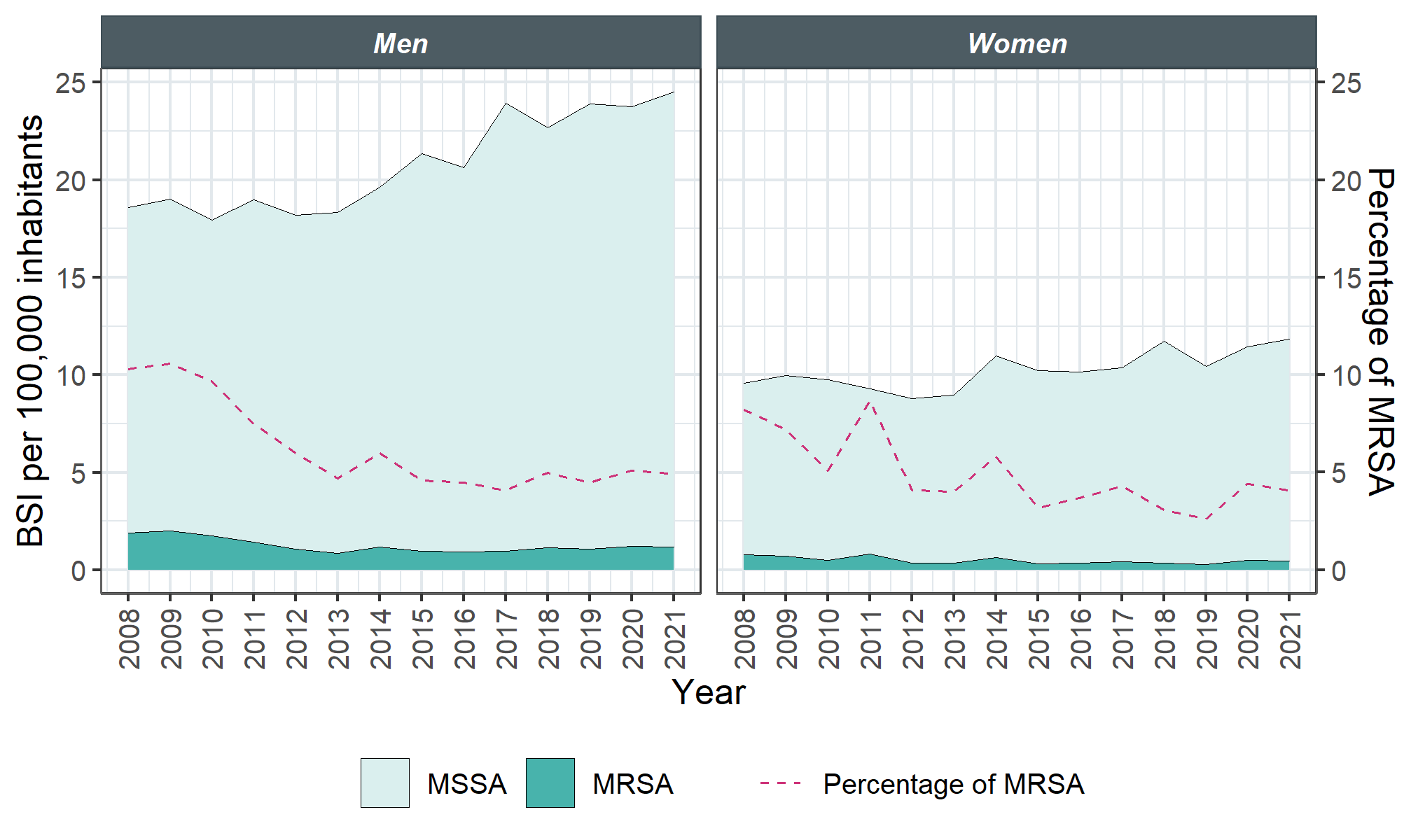


Supplementary Figure 3: Incidence in MSSA and MRSA bloodstream infections (BSI) and percentage of MRSA among *S. aureus* BSI stratified by sex (2008-2021)

_
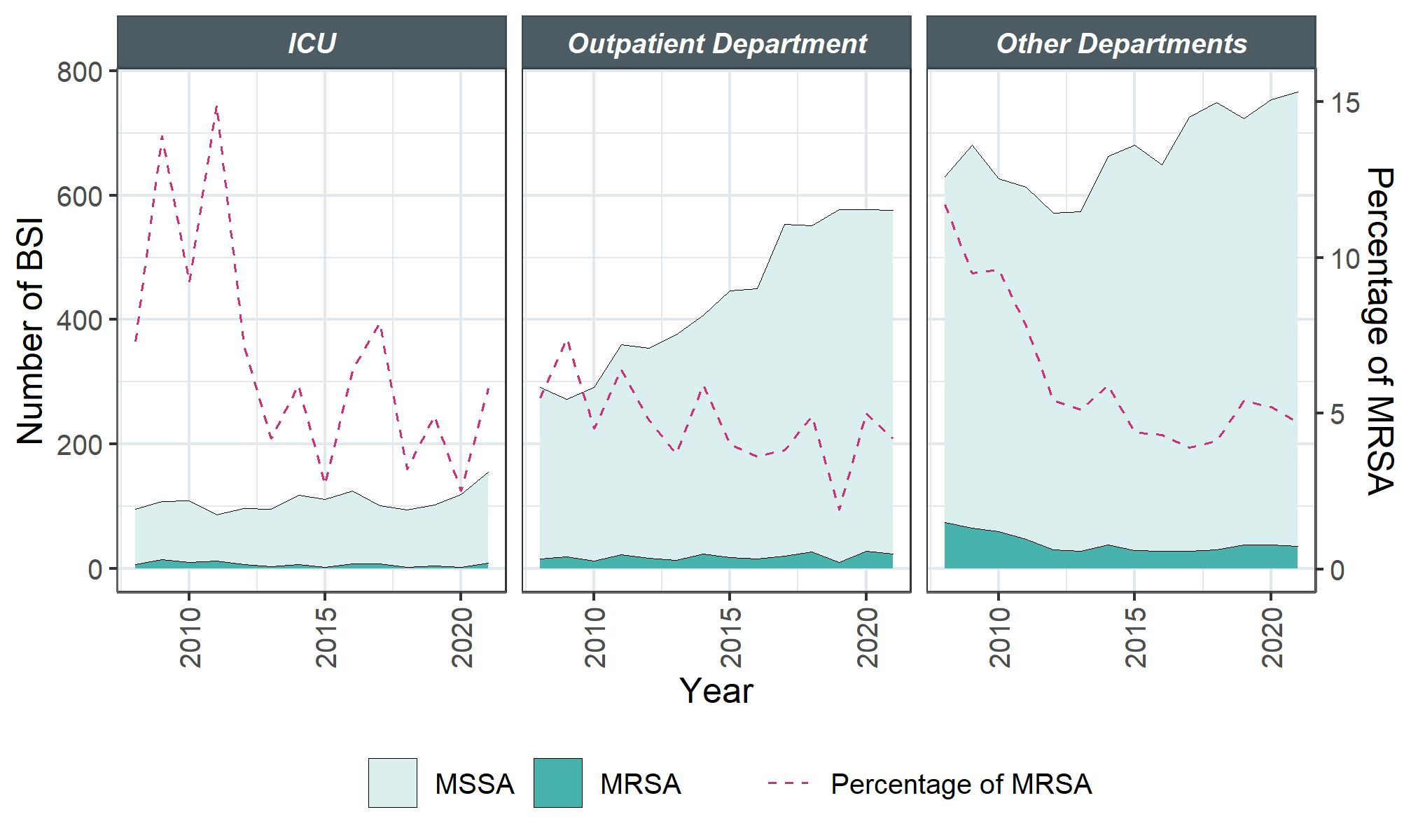
_

Supplementary Figure 4: Number of MSSA and MRSA bloodstream infections (BSI) and percentage of MRSA among *S. aureus* BSI stratified by hospital unit (2008-2021)


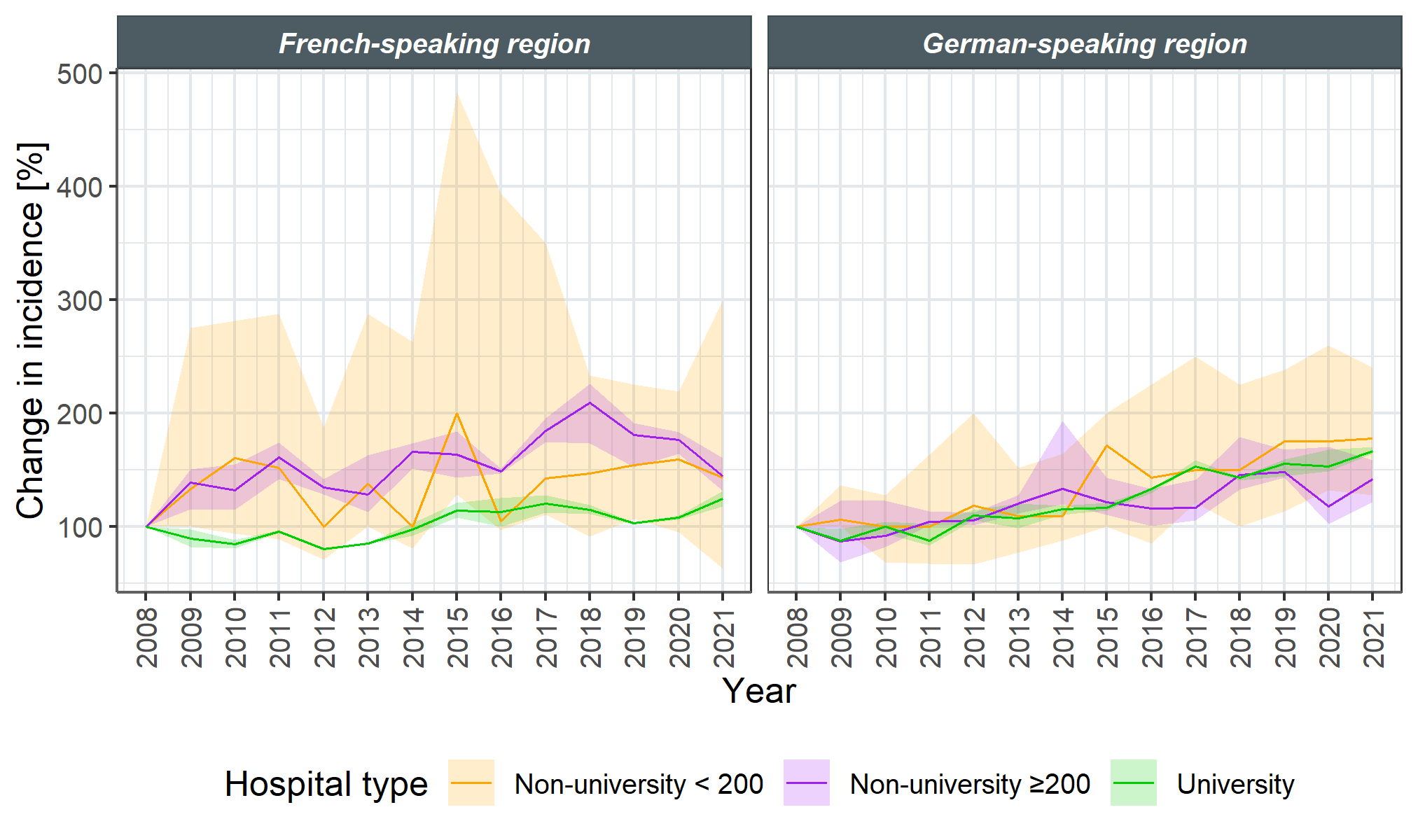


Supplementary Figure 5: Median (*bold line*) and interquartile range (*shaded area*) of proportional change in absolute numbers of MSSA BSI compared to 2008 grouped by hospital type and linguistic region (2008-2021)

MSSA BSI, methicillin-susceptible *Staphylococcus aureus* bloodstream infections


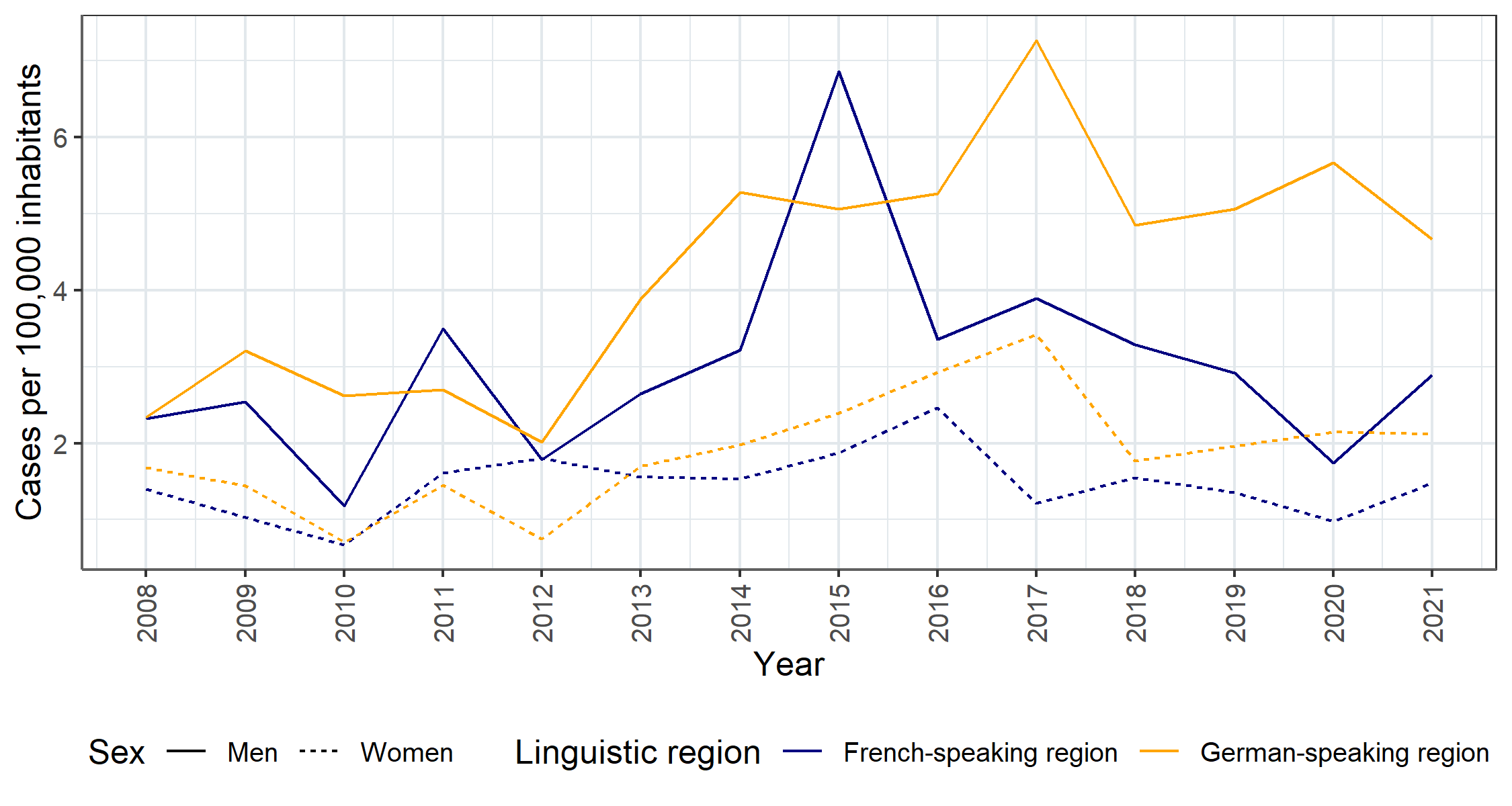


Supplementary Figure 6: Incidence of MSSA bone and joint infections stratified by linguistic region and sex (2008-2021)

MSSA, methicillin-susceptible *Staphylococcus aureus*


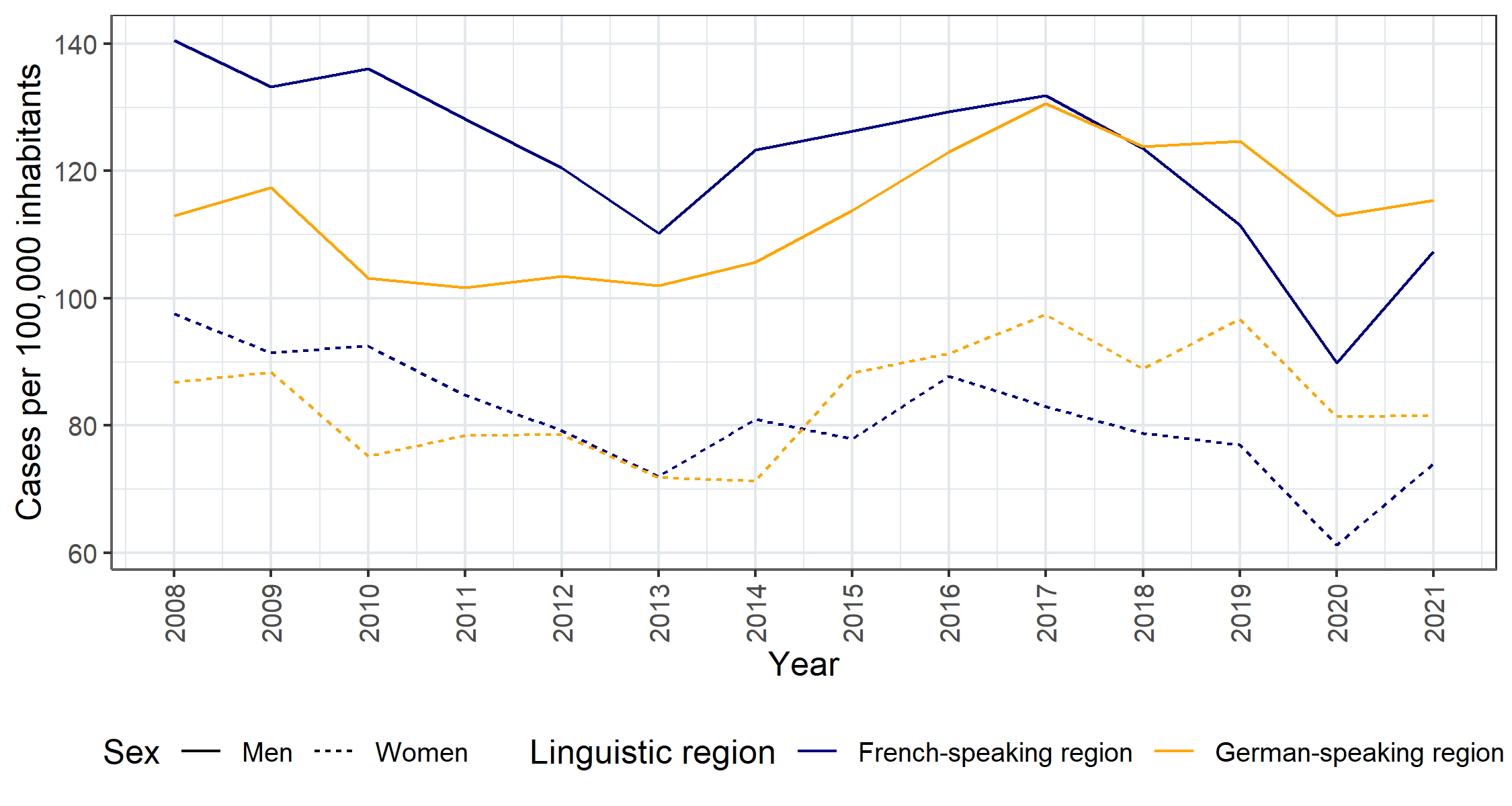


Supplementary Figure 7: Incidence of MSSA skin and soft tissue infections stratified by linguistic region and sex (2008-2021)

MSSA, methicillin-susceptible *Staphylococcus aureus*


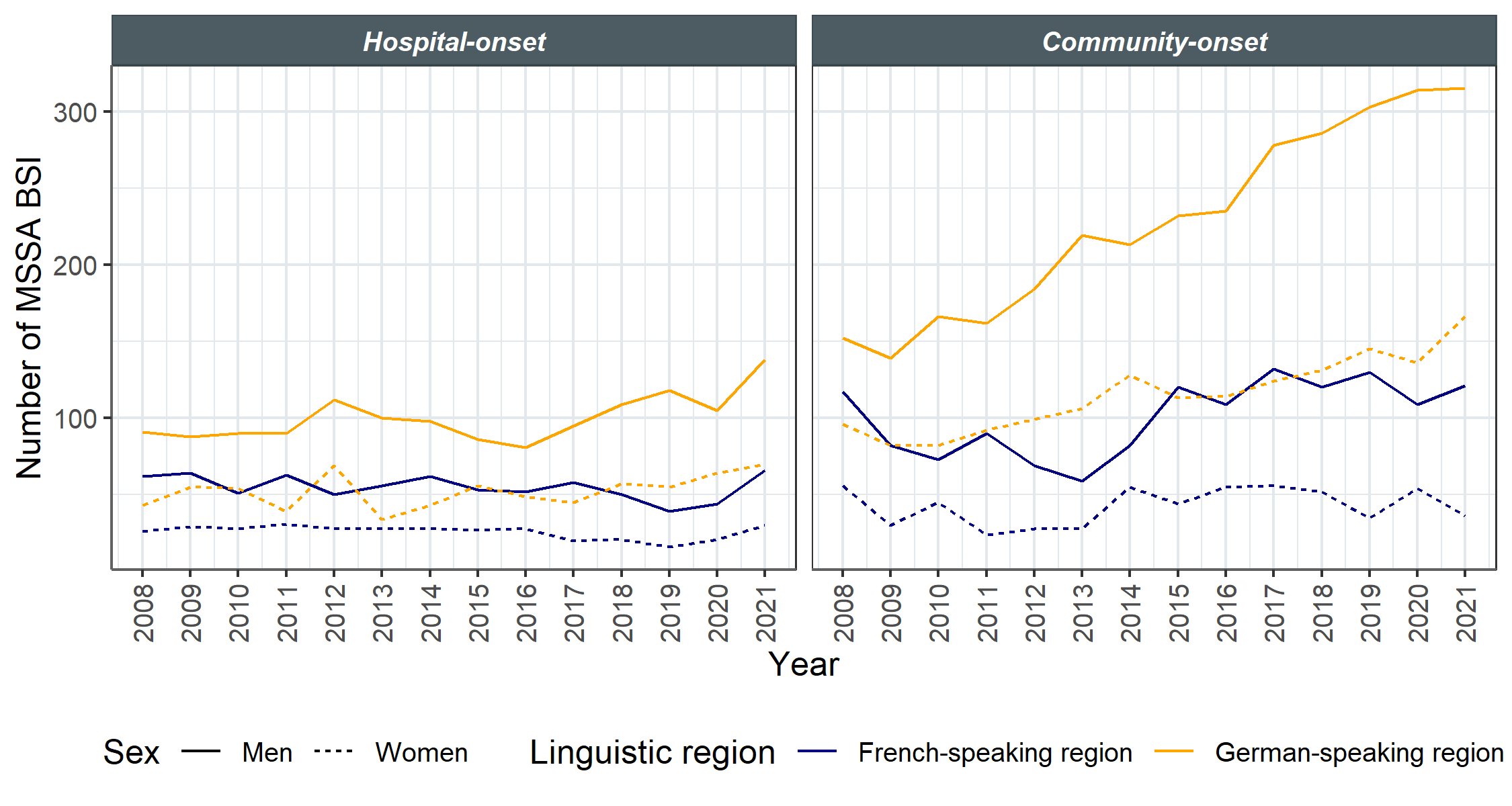


Supplementary Figure 8: Number of community-onset and hospital-onset MSSA BSI stratified by linguistic region and sex (2008-2021)

MSSA BSI, methicillin-susceptible *Staphylococcus aureus* bloodstream infections
